# Acute Mirror M1 Occlusions Treated With Endovascular First-Pass Contact Aspiration Technique

**DOI:** 10.7759/cureus.27350

**Published:** 2022-07-27

**Authors:** Evan Joyce, Brandon Sherrod, Jonathan P Scoville, Adam De Havenon, Ramesh Grandhi

**Affiliations:** 1 Department of Neurosurgery, University of Utah, Salt Lake City, USA; 2 Department of Neurology, University of Utah, Salt Lake City, USA

**Keywords:** thrombectomy, endovascular procedures, middle cerebral artery, infarction, stroke

## Abstract

Patients with acute bilateral M1 occlusion are usually comatose at presentation and have a grave prognosis. There have been few reports of emergent treatment using endovascular reperfusion therapy (ERT). We describe a patient treated with simultaneous first-pass contact aspiration and review the literature for cases describing the successful use of ERT in patients with bilateral anterior circulation proximal large-vessel occlusion (LVO). A functionally independent 95-year-old woman with history of atrial fibrillation (AF) presented with altered mentation, aphasia, and weakness in all extremities. Her National Institutes of Health Stroke Scale (NIHSS) score was 13. CT angiogram and perfusion demonstrated acute mirror M1 occlusions with extensive bilateral middle cerebral artery (MCA) territory penumbra, respectively. Emergent ERT was performed with simultaneous contact aspiration within the bilateral M1s under concomitant flow arrest with a balloon guide catheter. Modified Thrombolysis in Cerebral Infarction (mTICI) grades 3 and 2c were achieved on the left and right, respectively. By postoperative day 1 (POD1), the patient had improved motor function, mentation, and communication.The technical feasibility of simultaneous contact aspiration thrombectomies for acute bilateral M1 occlusions was demonstrated with successful reperfusion of both vascular territories in a single pass lasting 28 minutes. Simultaneous thrombectomies yielded rapid recanalization and reperfusion and minimized radiation exposure. Previous cases demonstrating this technique utilized stent-retriever techniques in successive fashion, with a consequent increase in the patient’s total ischemic time. The technical success of our aggressive approach suggests it may have utility in the treatment of acute multivessel occlusions (MVOs).

## Introduction

Over the last decade, endovascular reperfusion therapies (ERTs) have become standard of care in treating acute ischemic stroke (AIS) due to thromboembolic anterior circulation large-vessel occlusion (LVO) [1]. The majority of LVOs occur unilaterally in the proximal anterior cerebral vasculature in either the internal carotid artery (ICA) or the M1 segment of the middle cerebral artery (MCA) [1]. Thromboembolic multivessel occlusion (MVO), defined as concurrent acute occlusions either within or distant from an involved territory in patients undergoing ERT, occurs in about 10% of patients [2]. Occlusion of bilateral proximal MCAs is an even rarer subset of MVO estimated to account for less than 1% of cases of AIS [2,3].

Patients with acute bilateral M1 occlusion typically present comatose, and the diagnosis carries an extremely poor prognosis. To date, there have been two case reports of successful ERT with mechanical thrombectomy in these patients [3,4], while others have reported the use of ERT for MVOs involving bilateral proximal occlusion at the ICA or M1/2 in various combinations [5-9]. To the best of our knowledge, there have been no reports of technically successful simultaneous ERT using contact aspiration alone in acute bilateral M1 occlusions. Here, we present the first report of bilateral first-pass contact aspiration for acute mirror M1 occlusions.

## Case presentation

Presentation

A 95-year-old functionally independent woman presented to the emergency department with altered mentation, aphasia, and weakness in all extremities after being found down on the floor at home by family. She had a history of hyperlipidemia, hypertension, and atrial fibrillation (AF) and was taking rivaroxaban for anticoagulation. Her last known well was 14 hours earlier. On examination, she was awake and aphasic with rightward gaze deviation and was able to make weak but purposeful movement in all four extremities. Her National Institutes of Health Stroke Scale (NIHSS) score was 13 on admission.

Imaging

**Figure 1:**
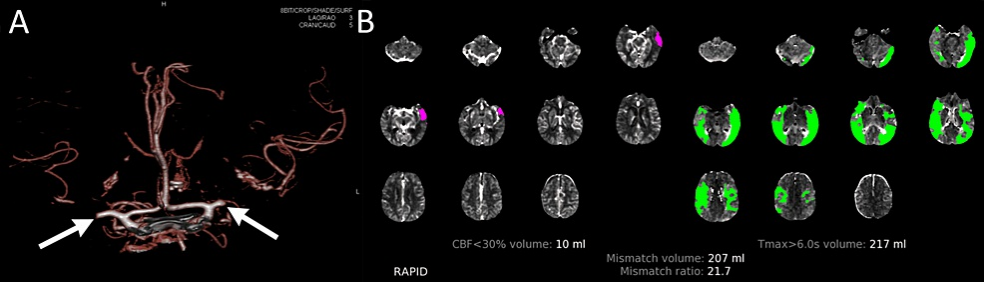
CT angiography and perfusion imaging (A) CT angiography demonstrated proximal middle cerebral artery (MCA) occlusions (arrows) with distal MCA-territory collaterals. (B) CT perfusion demonstrated large area of penumbra (green) with small anterior left temporal core infarct volume (pink).

CT demonstrated left MCA distribution early ischemic changes with Alberta Stroke Program Early CT Score (ASPECTS) of 8. CT angiography demonstrated bilateral, mirror proximal M1 MCA occlusions (Figure 1A), and CT perfusion showed extensive bilateral MCA distribution of penumbra of 217 ml with a small core infarct of 10 ml in the left anterior temporal lobe, yielding a penumbra:core mismatch ratio of 21.7 (Figure 1B). She did not receive recombinant intravenous tissue plasminogen activator (tPA) because she was outside the time window for administration. Her family wished to proceed with an emergent ERT for the possibility of language restoration and functional improvement given her significant penumbra on CT perfusion.

Treatment

**Figure 2:**
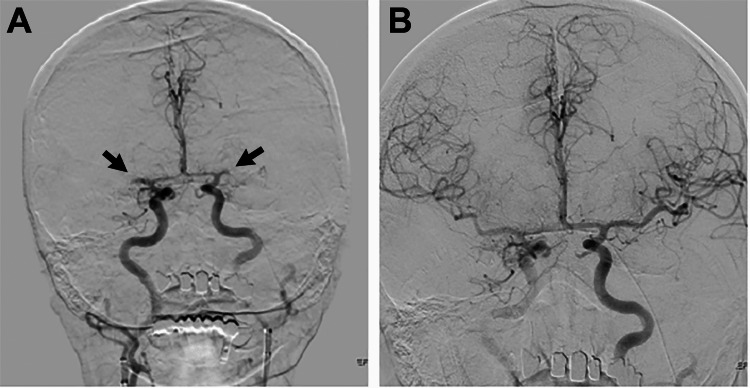
Cerebral digital subtraction angiography. (A) Bilateral ICAs showing acute occlusion at bilateral M1 segments. (B) Recanalization after first-pass contact aspiration under manual aspiration with flow arrest was TICI 3 on the left and 2c on the right.

The patient was brought to the endovascular suite for emergent ERT. 9F Pinnacle introducer sheaths (Terumo Medical Corp., Somerset, USA) were placed in the bilateral common femoral arteries, and 9F Cello balloon guide catheters (Medtronic, Irvine, USA) were subsequently advanced sequentially into the bilateral common carotid arteries. Under roadmap guidance, the balloon guide catheters were then advanced into the bilateral internal carotid arteries (ICAs). The first angiogram of the bilateral ICAs was performed simultaneously with two operators through the balloon guide catheters (Figure 2), clearly demonstrating mirror M1 occlusions (Figure 2A) with excellent pial collateralization from anterior cerebral artery to MCA circulation.

Under roadmap guidance, two operators simultaneously performed the next series of steps in unison. First, the clots were crossed with Fathom 0.016” steerable microwires (Boston Scientific, Malborough, USA) and VIA 27 microcatheters (Microvention, Aliso Viejo, USA). Next, a large-bore 0.071” aspiration catheter (Cerenovus, J&J, New Brunswick, USA) was advanced over each VIA microcatheter. Contact aspiration thrombectomy was performed simultaneously with manual aspiration through the large-bore aspiration catheters under concomitant anterior circulation flow arrest using the 9F Cello balloon guide catheters, which were positioned in the proximal petrous segment of the bilateral ICAs. The aspiration catheters were simultaneously retrieved with continuous manual aspiration until they were captured within the Cello catheters to prevent both distal and off-vessel embolization. The time elapsed from access to first-pass aspiration and from first angiogram to first-pass aspiration were 28 and eight minutes, respectively. After the first pass, there was modified Thrombolysis in Cerebral Infarction (mTICI) grade 3 recanalization on the left and grade 2c on the right (Figure 2B). On final intracranial angiography, both common femoral arteries were closed with 8F Angio-Seal devices (Terumo Medical Corp., Somerset, USA).

Postoperative course

**Figure 3:**
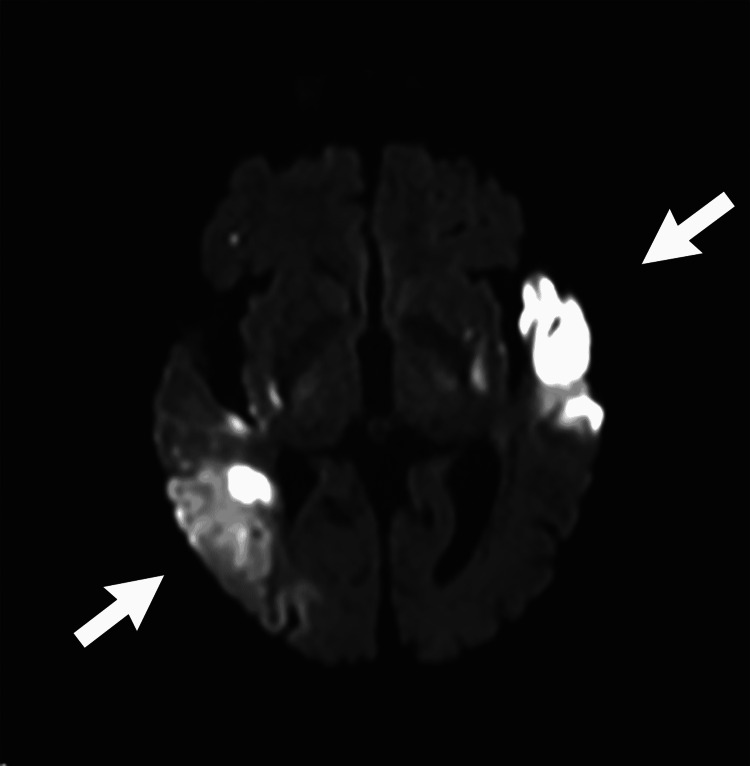
MRI showed areas of diffusion restriction in the left anterior temporal lobe, left basal ganglia, and right posterior temporoparietal region

There were no periprocedural complications. On postprocedure day 1, the patient had improved mentation, speech production, interaction with family, and near full strength in all extremities. MRI showed diffusion restriction in the left anterior temporal lobe, left basal ganglia, and right posterior parietotemporal cortex (Figure 3). However, the patient failed her swallow evaluation and was agitated and appeared uncomfortable with placement of a nasoduodenal feeding tube. Her family ultimately chose to transition her to comfort care, and she died six days after stroke ictus. 

## Discussion

Acute bilateral M1 occlusion is a rare type of MVO in AIS that requires prompt recognition and emergent treatment for meaningful clinical improvement. Here we describe the first reported case of technically successful treatment done with simultaneous first-pass contact aspiration under manual aspiration with anterior circulation flow arrest. We demonstrate the technical feasibility of this approach, with an access-to-recanalization time of 28 minutes and a first angiogram-to-recanalization time of 8 minutes. The use of two operators working in unison facilitated rapid simultaneous revascularization of the bilateral MCA territories.

In a study of 720 consecutive patients treated endovascularly for AIS, Kaesmacher et al. [2] found MVO in 10.7% of patients, with approximately two thirds of these occurring in different vascular territories. MVO is a challenging entity to treat and is associated with lower rates of reperfusion and good functional outcome [2]. Endovascular treatment of bilateral proximal cerebrovascular MVO is especially rare and technically challenging.

We reviewed the literature for cases describing the successful use of ERT in patients with bilateral anterior circulation proximal LVO and identified 9 previous reports (Table 1). Overall there were two reports with occlusion at bilateral M1 [3,4], two with M1 and contralateral M2 [6-9], three with M1 and contralateral ICA [5,6,9], and one with bilateral ICA [8]. With the inclusion of the present case, the average patient age was 75.3 years (range: 64-95) and the presenting NIHSS was 15.4 (range: 11-26; n=7). All strokes were presumed to be cardioembolic, with AF documented during hospitalization. Excluding three wake-up strokes, the average time since last known well was 5.3 hours. Three of five patients who presented within the intravenous thrombolysis time window (<4.5 hours) received tPA [4,9], as did one patient outside the window [6]. With the exception of the present case and the case reported by Larrew et al. [8], these reports all described the use of either a first-line stent retriever or a stent retriever with concomitant local aspiration, both of which are widely used, efficacious techniques for patients with LVO [10]. Aside from the present case, one other case involved a technique of simultaneous thrombectomy [8]. Technical success (i.e., recanalization with mTICI≥2b) was achieved in 95% (19/20) of the vessels treated among these reports, and overall, half the patients (5/10) achieved a good functional outcome (i.e., modified Rankin Scale (mRS)≤2). Including our patient, there were three deaths, all as a result of family withdrawing care early in the postoperative period [6,8].

**Table 1:** Literature review of reported ERT for bilateral acute proximal vessel MVO ICA: internal carotid artery; NIHSS = National Institutes of Health Stroke Scale; LKW = last known well; tPA = tissue plasminogen activator; ERT = endovascular reperfusion therapy; mTICI = modified Thrombolysis in Cerebral Infarction; mRS: modified Rankin scale; F/u = follow-up; AF = atrial fibrillation; POD = postoperative day; NR = not reported *with concurrent flow reversal **also left distal P2 occlusion without ERT attempt

Study	Age (years)/ Sex	NIHSS, LKW	tPA	Etiology	CT-P	Vessels (L/R)	Stent-retriever vs. contact aspiration	mTICIs (L/R)	Simultaneous	Complications	F/u
Pop et al. 2014 [9]	78F	26, 3.5 hr	Y	AF; cardioembolic	N	ICA/M2	stent-retriever*	3/2b	N	N	NIHSS 0 at 30 days
	66F	18, 3.5 hr	Y	AF; cardioembolic	N	M1/ICA	stent-retriever*	3/2b	N	N	NIHSS 8, mRS 3 at three months
Dietrich et al., 2014 [5]	72M	NR, 3 h	N	AF; cardioembolic	N	ICA/M1	stent-retriever	3/3	N	N	Rehab on POD14 with “minor neurological symptoms”
Ramos et al., 2018 [6]	68F	12, 18 h	N	AF; cardioembolic	Y	M1/M2	stent-retriever	3/1	N	Unilateral hemorrhagic transformation	mRS 4 at three months
	82F	16, 6 h	Y	NR	N	M1/ICA	NR	3/3	N	N	Death on POD10
Storey et al., 2019 [7]	64F	NR, wake up	N	AF; cardioembolic	Y	M2/M1	stent-retriever with contact aspiration	3/3	N	N	Rehab on POD3, “following commands with aphasia resolved”
Larrew et al., 2019 [8]	NR, M	NR, 1 hr	N	Acute MI; cardioembolic	Y	ICA/ICA	contact aspiration	2b/3	Y	N	Family withdraw, death
Heyworth et al., 2020 [3]	69F	12, wake up	N	AF; cardioembolic	Y	M1/M1	stent-retriever with contact aspiration	3/3	N	N	NIHSS 0 on POD3
London et al., 2020 [4]	84F	11, 2.5 hr	Y	AF; cardioembolic	N	M1/M1**	stent-retriever with contact aspiration	3/3	N	N	NIHSS 0 on POD1, mRS 1 at 90 days
This paper	95F	13, wake up	N	AF; cardioembolic	Y	M1/M1	contact aspiration	3/2c	Y	N	Family withdraw, death on POD6

Within our reviewed cohort there were two other published reports of successful ERT for acute bilateral M1 occlusion [3, 4]. Both cases involved a non-simultaneous approach in which the operators utilized a stent retriever with local aspiration. Although London et al. [4] started with a direct, first-pass technique (ADAPT) at the right M1, they required the use of a stent retriever with concomitant local aspiration for thrombectomy. In both cases, the authors achieved bilateral mTICI grade 3 revascularization and the patients ultimately achieved a good functional outcome with postoperative NIHSS of 0 [3, 4].

When the MVO affecting proximal vessels of different vascular territories is identified before the procedure, we advocate using a simultaneous first-pass contact aspiration technique (e.g., ADAPT). In the present case, two surgeons worked in unison to advance the balloon guide catheters into the ICAs and subsequently cross the M1 clots and perform manual aspiration for thrombectomy under a single roadmap. Using two surgeons decreased time to revascularization of bilateral MCA territories while minimizing radiation exposure. In our experience, this did not add undue complexity. Clear and concise communication was imperative in this approach.

Contact aspiration is a highly effective method for mechanical thrombectomy, particularly in the proximal MCA [11]. The results of the ASTER trial showed no difference in successful revascularization rate or number of revascularization attempts when comparing contact aspiration to stent retriever use for mechanical thrombectomy. Moreover, the time to revascularization appeared to be somewhat shorter associated with contact aspiration compared with stent retriever thrombectomy (38 vs. 45 minutes, p<0.10) [12]. One could reasonably expect this time difference to further increase if two occlusions were treated successively in different anatomical locations.

Larrew et al. [8] reported a similar approach to ours with bilateral groin access for simultaneous contact aspiration thrombectomies in the setting of bilateral ICA terminus MVO. Their time to recanalization of 32 minutes was similar to that achieved in our case. Simultaneously treating the occlusions with bilateral groin access and dual catheter manipulation, while perhaps presenting more procedural complexity, likely improved recanalization time and minimized radiation exposure to operator and patient. In the present case, two surgeons worked in unison to advance the balloon guide catheters, cross the M1 clots, and perform manual aspiration for thrombectomy. However, concurrent flow arrest was not used in their case, and they noted multiple downstream distal emboli in major branches of each ICA territory. These distal emboli required additional aspiration passes at the bilateral A1 and M1 segments to achieve satisfactory reperfusion. Our approach of achieving complete anterior circulation flow arrest with balloon guide catheter inflation in the petrous ICA segment was safe and may have helped mitigate downstream emboli.

Although our patient ultimately died as a result of her stroke, the decision to pursue emergent ERT was guided by the extensive bilateral MCA penumbra and mismatch delineated by her perfusion imaging. Preoperatively, her imaging demonstrated a small core infarct in the left anterior temporal lobe, but there was significant potential to improve her clinical outcome with rapid flow restoration. In our reviewed cohort of patients undergoing ERT for MVO who were outside the early reperfusion therapy window (i.e., >6 hours), 100% had pre-procedure CT perfusion imaging. The DEFUSE 3 trial demonstrated ERT to be safe and effective out to 16 hours when guided by perfusion imaging in cases with perfusion mismatch [13]. Subgroup analysis found ERT benefits persist regardless of patient age, presenting NIHSS score, and time to randomization, when guided by perfusion imaging [14]. Others have found that, although outcomes are inferior to those of younger patients, elderly patients still have significant benefit from ERT, with at least 1 in 4 octogenarians regaining functional independence by 3 months [15].

## Conclusions

Acute bilateral M1 occlusion is a rare subset of ischemic stroke with a poor prognosis. To date, there remain few reported cases treated with ERT. We present the first report of simultaneous first-pass contact aspiration under manual aspiration with flow arrest that expeditiously achieved a technically successful outcome. This simultaneous technique holds potential to achieve rapid revascularization for AIS caused by MVO.
